# Phenotypic Classification of Scalp High-Frequency Oscillations in Absence Epilepsy Based on Multiple Characteristics Using K-Means Clustering

**DOI:** 10.3390/bioengineering13010065

**Published:** 2026-01-07

**Authors:** Keisuke Maeda, Himari Tsuboi, Nami Hosoda, Junichi Fukumoto, Shiho Fujita, Shunta Yamaguchi, Naohiro Ichino, Keisuke Osakabe, Keiko Sugimoto, Gen Furukawa, Naoko Ishihara

**Affiliations:** 1 Department of Clinical Physiology, Fujita Health University School of Medical Sciences, 1-98 Dengakugakubo, Kutsukake-cho, Toyoake 470-1192, Japan; 2 Department of Clinical Laboratory, Fujita Health University Hospital, 1-98 Dengakugakubo, Kutsukake-cho, Toyoake 470-1192, Japan; 3 Department of Medical Sciences Education, Fujita Health University School of Medical Sciences, 1-98 Dengakugakubo, Kutsukake-cho, Toyoake 470-1192, Japan; 4 Department of Pediatrics, Fujita Health University School of Medicine, 1-98 Dengakugakubo, Kutsukake-cho, Toyoake 470-1192, Japan

**Keywords:** high-frequency oscillations, absence epilepsy, k-means clustering analysis, biomarker, electroencephalography

## Abstract

Scalp high-frequency oscillations (HFOs) are promising noninvasive biomarkers of epileptogenicity, but their phenotypic diversity and clinical relevance in absence epilepsy (AE) remain unclear. This study aimed to classify scalp HFOs in AE using k-means clustering based on multiple morphological characteristics, and to evaluate their distribution across electroencephalogram (EEG) epochs and seizure control statuses. We analyzed scalp EEG recordings from 14 children and adolescents with AE. After excluding outliers, 163 scalp HFOs were characterized by average frequency, duration, amplitude, and number of cycles. Amplitude and cycle count were log-transformed prior to clustering, and k-means clustering was applied to identify distinct HFO phenotypes. Three clusters were identified: Cluster 1 (short duration, low amplitude), Cluster 2 (low frequency), and Cluster 3 (long duration, high cycle count). Cluster 2 and Cluster 3 were significant predictors of ictal HFOs in active AE, with odds ratios (ORs) of 0.33 (95% confidence interval [CI]: 0.14–0.74) and 5.00 (CI: 2.02–17.73), respectively. Cluster 2 also predicted interictal HFOs in active AE (OR [95% CI] = 2.71 [1.23–5.67]). These findings support the utility of scalp HFO phenotypes as EEG-based biomarkers for seizure detection and disease monitoring, potentially guiding treatment strategies in pediatric AE.

## 1. Introduction

Absence epilepsy (AE) is the most prevalent subtype of pediatric epilepsy, accounting for approximately 10–17% of childhood-onset cases [1]. AE is clinically characterized by absence seizures—brief, nonconvulsive episodes marked by staring and a sudden loss of responsiveness [2,3,4]. Although such seizures are easily recognized, their clinical presentation varies considerably between individuals [5]. Ictal electroencephalogram (EEG) recordings typically show regular, generalized spike-and-wave discharges at 3 Hz (range: 2.5–4 Hz) within the first second of seizure onset [5,6]. Electroclinical characterization of EEG patterns in AE is crucial for accurate diagnosis, tailored anti-seizure medication (ASM) selection, and prognostic evaluation.

Traditionally, EEG interpretation has been limited to the Berger frequency band (1–25 Hz). However, advances in recording techniques now allow analysis beyond this range [7]. High-frequency oscillations (HFOs), defined as EEG activity above 80 Hz, have emerged as promising biomarkers for epileptogenicity [8]. Initially observed via intracranial EEG recordings, HFOs have been identified as reliable biomarkers of epileptogenicity [9]. Recent studies have demonstrated the feasibility of noninvasive HFO detection from scalp EEG recordings, suggesting their potential as biomarkers for predicting epilepsy prognosis in pediatric populations [10,11,12,13]. Scalp-recorded HFOs (scalp HFOs) have attracted increasing attention for their utility in assessing seizure risk [14], disease severity [15,16], and treatment response [17,18,19]. In patients with AE, HFOs have been reported during both ictal and interictal EEG epochs [20,21]. However, the relationship between scalp HFO characteristics and seizure control status remains under-investigated. Further exploration of scalp HFO characteristics could enhance their clinical applicability and serve as a useful tool for identifying patients at risk of poor outcomes.

Recent developments in automated HFO classification have introduced deep learning-based approaches to improve detection accuracy and reduce reliance on manual annotation. Zhang et al. (2022) developed a reverse engineering framework using intracranial EEG data to distinguish epileptogenic HFOs from non-epileptogenic ones, achieving high classification performance and demonstrating clinical relevance [22]. Krikid et al. (2024) proposed a novel convolutional neural network (CNN) architecture for multi-classification of HFOs in intracranial EEG signals, using time–frequency representations and data augmentation to enhance generalization. Their results demonstrated superior performance in distinguishing HFOs from interictal epileptic spikes [23]. More recently, Chaibi and Kachouri (2025) introduced a novel deep learning framework combining LSTM and OWL-ViT models to distinguish epileptic HFOs from artifacts with high accuracy [24]. Ding et al. (2025) developed PyHFO 2.0, an open-source platform integrating deep learning models for clinical HFO analysis [25]. These studies highlight the growing interest in automated approaches to HFO detection and classification.

In this study, we performed k-means clustering on scalp HFOs detected from EEG recordings of patients with AE, including both ictal and interictal epochs. Clustering was based on four key characteristics: average frequency, duration, amplitude, and number of cycles. K-means clustering, a widely utilized unsupervised algorithm, facilitates the classification of unlabeled data into meaningful groups [26]. Next, we hypothesized that clusters based on HFO characteristics would exhibit different distributions across three EEG epochs: the ictal and interictal epochs in active AE and the interictal epoch in seizure-free AE. We then compared how the classified clusters were distributed across the three epochs in the EEG recordings and evaluated the predictive ability of the three epochs using the clusters. Finally, we assessed differences in scalp HFO characteristics among the three EEG epochs. Although various clustering algorithms have been proposed in recent literature, we selected k-means clustering for its interpretability, simplicity, and suitability for our data structure. The four HFO characteristics used—average frequency, duration, amplitude, and number of cycles—are continuous variables that align well with Euclidean distance-based clustering. K-means clustering also facilitates clear visualization of cluster centroids, which is beneficial for clinical interpretation. Importantly, while k-means itself is not novel, our study is among the first to apply it to scalp-recorded HFOs in absence epilepsy, a context that has received limited attention. Exploring these characteristics could help identify EEG markers useful for guiding prognosis.

## 2. Results

### 2.1. Basic Characteristics of the 14 Patients

Table 1 summarizes the basic characteristics of the patients with AE, stratified by seizure control status: active AE and seizure-free AE. Of the 14 patients included in the study, eight and six were classified as having active and seizure-free AE, respectively.

**Figure 1:**
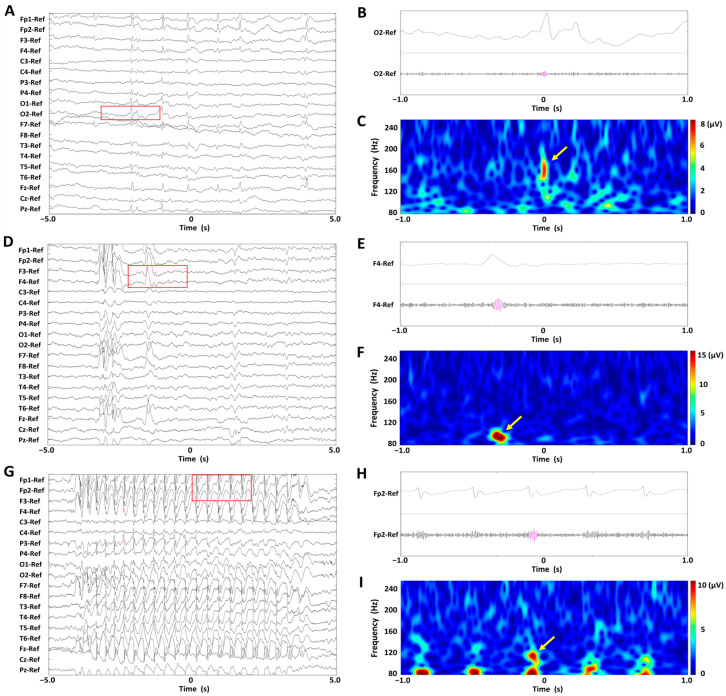
Representative EEG waveforms from three EEG epochs: ictal in active absence epilepsy (AE), interictal in active AE, and interictal in seizure-free AE. Each panel includes an example of a detected high-frequency oscillation (HFO). (**A**–**C**) Interictal epoch in seizure-free absence epilepsy (AE). (**D**–**F**) Interictal epoch in active AE. (**G**–**I**) Ictal epoch in active AE. (**A**,**D**,**G**) Unfiltered EEG recordings with normal time scales. (**B**,**E**,**H**) Lead signals with extended time scales (highlighted by red rectangles) and bandpass-filtered EEG recordings at 80–250 Hz. (**C**,**F**,**I**) Spectrograms of the EEG data corresponding to the red rectangles, showing time–frequency analysis. Scalp high-frequency oscillations (HFOs) are visible in both unfiltered and filtered EEG traces (pink overlays) and correspond to spectral blobs in the time–frequency plots (yellow arrows). Abbreviations: AE, absence epilepsy; HFO, high-frequency oscillation.

Among the five male patients, two had active AE and three had seizure-free AE. Among the nine female patients, six had active AE and three had seizure-free AE. The mean (standard deviation (SD)) ages at the time of the EEG recording and AE onset were 9.0 (4.2) and 6.5 (1.3) years, respectively, for patients with active AE, and 14.2 (3.0) years and 8.2 (2.9) years, respectively, for those with seizure-free AE. Age and sex distributions were balanced across active and seizure-free AE groups, minimizing potential confounding effects in subsequent analyses. Of the 12 patients diagnosed with CAE, eight had active AE and four had seizure-free AE. The remaining two patients were diagnosed with JAE, both of whom were classified as seizure-free AE. During the interictal EEG epochs, 71 scalp HFOs were detected in patients with active AE, and 35 in those with seizure-free AE. Additionally, in the ictal EEG epochs of patients with active AE, 57 scalp HFOs were identified. Representative EEG waveforms corresponding to each EEG epoch—ictal in active AE, interictal in active AE, and interictal in seizure-free AE—are shown in Figure 1, along with examples of detected HFOs.

### 2.2. K-Means Clustering Analysis of Scalp HFOs Based on HFO Characteristics

A total of 163 scalp HFOs detected from patients were classified using k-means clustering analysis based on four characteristics: average frequency, duration, and the log-transformed amplitude and number of cycles. The optimal clustering solution identified three distinct clusters—Cluster 1, Cluster 2, and Cluster 3—comprising 65, 70, and 28 scalp HFOs, respectively.

**Figure 2:**
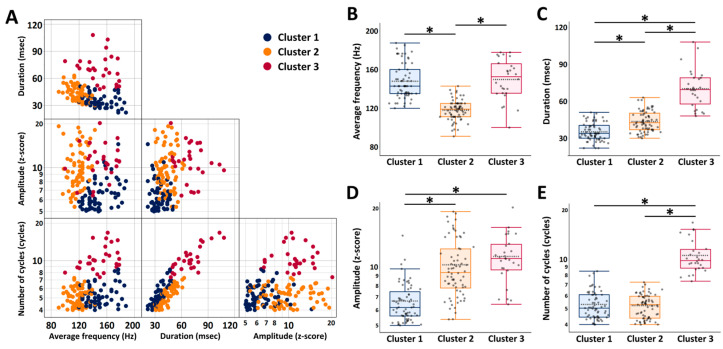
Cluster analysis of scalp high-frequency oscillations based on four morphological characteristics and comparisons across clusters. (**A**) Principal component plot of scalp HFOs classified by k-means clustering into Cluster 1 (blue), Cluster 2 (yellow), and Cluster 3 (red). Each point is projected onto a two-dimensional space defined by two of the four HFO characteristics. (**B**–**E**) Boxplots comparing scalp HFO characteristics among the three clusters: Cluster 1 (*n* = 65), Cluster 2 (*n* = 70), and Cluster 3 (*n* = 28). Boxplots display the median (bold horizontal line), mean (horizontal dotted line), interquartile range (box), and non-outlier range (whiskers). * *p*  <  0.001 (Tukey–Kramer honestly significant difference [HSD] tests). Since the amplitude and number of cycles had log-normal distributions, log-transformed values were used for the analyses. Abbreviations: HFO, high-frequency oscillation; HSD, honestly significant difference.

The results of the three-cluster solution are illustrated in Figure 2. The distribution of scalp HFOs across average frequency, duration, amplitude, and number of cycles is shown in Figure 2A. Comparative analyses of these characteristics between clusters are presented in Figure 2B–E. Cluster 1 exhibited the shortest durations (mean ± SD: 35.3 ± 7.3 ms) and the lowest amplitudes (median [interquartile range (IQR)] z-score: 6.2 [5.6–7.4]). Cluster 2 had the lowest average frequency (mean ± SD: 118.1 ± 10.5 Hz). Cluster 3 showed the longest durations (mean ± SD: 70.2 ± 15.4 ms) and the highest number of cycles (median [IQR]: 9.8 [8.8–11.5] cycles).

### 2.3. Multinomial Logistic Regression Analysis of Cluster Groups as Predictors of EEG Epochs: Ictal and Interictal Epochs in Active AE, and Interictal Epoch in Seizure-Free AE

We hypothesized that clusters classified based on HFO characteristics would exhibit distinct distributions across three EEG epochs: (1) the ictal epoch in active AE, (2) the interictal epoch in active AE, and (3) the interictal epoch in seizure-free AE. A comparison of the proportions of these epochs across the three clusters revealed significant differences (χ^2^  =  66.4, *p*  <  0.001). Cluster 2 showed a tendency toward a higher proportion of scalp HFOs detected during the interictal epoch in active AE, while Cluster 3 showed a higher proportion of scalp HFOs detected during the ictal epoch in active AE (Table 2).

The results of the multinomial logistic regression analysis, using the interictal epoch in seizure-free AE as the reference category, are presented in Table 2. A significantly higher odds ratio (OR) for the interictal epoch in active AE was observed in Cluster 2 (OR [95% confidence interval (CI)]  =  2.71 [1.23–5.67], *p*  =  0.009) compared with Cluster 1. Compared with Cluster 1, the OR for ictal epoch in active AE was significantly higher in Cluster 3 (OR [95% CI]  =  5.00 [2.02–17.73], *p*  =  0.002) and significantly lower in Cluster 2 (0.33 [0.14–0.74], *p*  =  0.009). These findings indicate that Cluster 2 and Cluster 3 were significant predictors of HFO occurrence during the ictal epoch in active AE, while Cluster 2 was a significant predictor of HFO occurrence during the interictal epoch in active AE.

### 2.4. Comparison of Scalp HFO Characteristics Among EEG Epochs: Ictal and Interictal Epochs in AE, and Interictal Epoch in Seizure-Free AE

**Figure 3:**
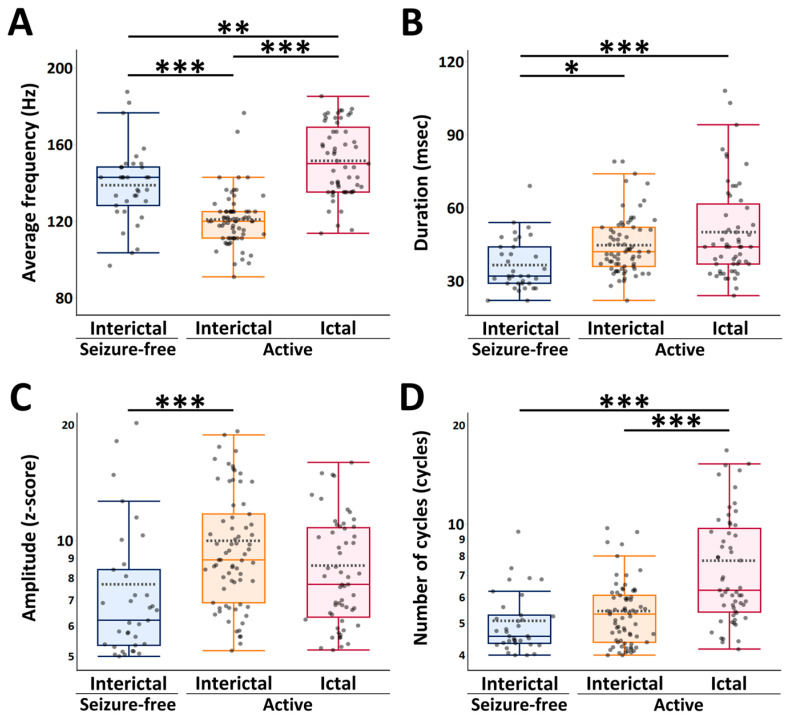
Comparison of scalp high-frequency oscillation characteristics across three electroencephalogram epochs: the ictal and interictal epochs in active absence epilepsy (AE), and the interictal epoch in seizure-free AE. Boxplots display the distribution of scalp HFO characteristics across the three EEG epochs. Each boxplot shows the median (bold horizontal line), mean (horizontal dotted line), interquartile range (box), and non-outlier range (whiskers). (**A**) Average frequency, (**B**) Duration, (**C**) Amplitude, and (**D**) Number of cycles. * *p*  <  0.05, ** *p*  <  0.01, *** *p*  <  0.001 (Tukey–Kramer honestly significant difference [HSD] tests). Since the amplitude and number of cycles had log-normal distributions, log-transformed values were used for the analyses. Abbreviations: AE, absence epilepsy; HFO, high-frequency oscillation; HSD, honestly significant difference.

Figure 3 presents the mean and median values of the scalp HFO characteristics detected across three EEG epochs: the ictal and interictal epochs in active AE, and the interictal epoch in seizure-free AE.

The mean average frequency of scalp HFOs detected during the interictal epoch in active AE (120.9 ± 13.8 Hz) was significantly lower than those observed during both the interictal epoch in seizure-free AE (138.8 ± 19.7 Hz, *p*  <  0.001) and the ictal epoch in active AE (151.5 ± 18.7 Hz, *p*  <  0.001). Additionally, the mean frequency during the interictal epoch in seizure-free AE was significantly lower than that during the ictal epoch in active AE (*p*  =  0.002). The mean duration of scalp HFOs detected during the interictal epoch in seizure-free AE (36.5 ± 10.6 ms) was significantly shorter than those during both the interictal epoch in active AE (44.7 ± 12.0 ms, *p*  =  0.02) and the ictal epoch in active AE (50.0 ± 19.2 ms, *p*  <  0.001). The median amplitude of scalp HFOs detected during the interictal epoch in seizure-free AE (z-score: 6.2 [IQR: 5.3–8.4]) was significantly lower than that during the interictal epoch in active AE (z-score: 8.9 [6.9–11.7], *p*  <  0.001). The median number of cycles of scalp HFOs detected during the ictal epoch in active AE (6.3 [5.4–9.7] cycles) was significantly higher than those during both the interictal epoch in active AE (5.3 [4.4–6.1] cycles, *p*  <  0.001) and the interictal epoch in seizure-free AE (4.6 [4.4–5.3] cycles, *p*  <  0.001).

### 2.5. Multiple Linear Regression Analysis of Scalp HFO Characteristics Across EEG Epochs: Ictal and Interictal Epochs in AE, and Interictal Epoch in Seizure-Free AE

Table 3 summarizes the associations between scalp HFO characteristics and three EEG epochs: the ictal and interictal epochs in active AE, and the interictal epoch in seizure-free AE. Statistical analyses were conducted using multiple linear regression, with the interictal epoch in seizure-free AE set as the reference category. In the unadjusted (crude) regression analysis, both the scalp HFO average frequency and the log-transformed number of cycles were significantly lower during the interictal epoch in active AE (average frequency: *β* [95% CI]  =  −16.19 [−19.78 to −12.61], *p*  <  0.001; log number of cycles: *β*  =  −0.08 [−0.14 to −0.02], *p*  =  0.008). The scalp HFO log-transformed amplitude was significantly higher during the interictal epoch in active AE (*β*  =  0.14 [0.07 to 0.21], *p*  <  0.001). During the ictal epoch in active AE, the scalp HFO average frequency, duration, and log number of cycles were all significantly higher (average frequency: *β*  =  14.43 [10.68 to 18.19]; duration: *β*  =  6.26 [3.01 to 9.52]; log number of cycles: *β*  =  0.23 [0.16 to 0.29]; all *p*  <  0.001).

In the regression analysis adjusted for age at the time of the EEG recording, scalp HFO average frequency remained significantly lower during the interictal epoch in active AE (*β* [95% CI]  =  −16.05 [−21.56 to −10.55], *p*  <  0.001). The scalp HFO log-transformed amplitude was significantly higher during the interictal epoch in active AE (*β*  =  0.19 [0.08 to 0.30], *p*  <  0.001). During the ictal epoch in active AE, the scalp HFO average frequency, duration, and log number of cycles were all significantly higher (average frequency: *β*  =  14.58 [8.76 to 20.41], *p*  <  0.001; duration: *β*  =  5.79 [0.75 to 10.84], *p*  =  0.025; log number of cycles: *β*  =  0.22 [0.13 to 0.32], *p*  <  0.001).

## 3. Discussion

The characteristics of HFOs have traditionally been described primarily in terms of frequency. In the present study, we expanded the feature space to include four key characteristics—average frequency, duration, amplitude, and number of cycles—and classified scalp HFOs using k-means clustering analysis based on these multidimensional characteristics. This approach yielded three distinct clusters. Most previous studies applying clustering or deep learning techniques to HFO analysis have focused on intracranial EEG data, which, while informative, require invasive procedures. In contrast, our study demonstrates the utility of k-means clustering applied to HFOs detected from scalp EEG recordings. This non-invasive approach is particularly relevant for pediatric populations, where invasive monitoring is less feasible. By characterizing scalp HFO phenotypes and linking them to seizure states, our findings offer a clinically applicable framework for EEG-based biomarker development in absence epilepsy. For example, Cluster 3, which was strongly associated with ictal activity, could be used to support real-time seizure detection or to monitor treatment efficacy. Cluster 2, associated with interictal activity in active AE, may help identify patients at risk of poor seizure control or relapse. Furthermore, the phenotypic classification of HFOs may contribute to individualized treatment strategies by providing electrophysiological markers that reflect disease activity. Future studies could explore the integration of these HFO profiles into longitudinal monitoring systems or predictive models for seizure recurrence.

Cluster 3 was characterized by scalp HFOs with the longest duration and highest cycle count, and was predominantly observed during the ictal epochs in active AE. By contrast, Cluster 3 was rarely detected during the interictal epochs in either active or seizure-free AE. These findings suggest that Cluster 3 represents ictal HFOs associated with absence seizures, and its profile may serve as a useful marker for identifying seizure-related activity. To date, few studies have focused on ictal HFOs, with those that have primarily having examined focal epilepsies [27,28]. Therefore, our characterization of ictal HFOs in patients with AE offers a novel contribution to the field.

Cluster 2 was defined by scalp HFOs with the lowest average frequency, and was frequently observed during the interictal epochs in active AE. It was rarely detected during the ictal epochs in active AE or the interictal epochs in seizure-free AE. Cluster 1, characterized by the shortest duration and lowest amplitude, was observed at similar rates across EEG recordings from both patients with active and seizure-free AE. Given its association with active AE, Cluster 2 may represent pathological HFOs, whereas Cluster 1 may reflect physiological HFOs.

Physiological (non-epileptic) HFOs were first identified in the ripple band of the hippocampus in healthy rats [29,30], and later observed in the non-epileptic sensorimotor cortex of children with focal epilepsy [31]. Traditionally, HFOs have been classified as epileptogenic or physiological based on detection rate thresholds [32,33]. However, detection rates vary by anatomical region [34] and can be elevated even in normal brain areas [35]. Beyond the detection rate, morphological features such as amplitude, duration, and frequency have also been investigated to distinguish physiological from pathological HFOs, though findings have been inconsistent [33,36,37,38]. Our results provide new insights into the morphological distinctions between pathological and physiological scalp HFOs in AE. Further research is warranted to determine which types of epilepsy and seizure states are most amenable to prediction based on the three scalp HFO clusters identified in the present study.

Given the coexistence of both physiological and pathological HFOs, it is essential to delineate their distinguishing characteristics to advance the use of pathological HFOs as reliable biomarkers for epilepsy [39]. Our findings demonstrated that scalp HFOs observed in active AE exhibited lower average frequency, longer duration, and higher amplitude compared with those in seizure-free AE. These results are consistent with previous studies. Matsumoto et al. [37] reported that pathological HFOs tend to have higher spectral amplitude and longer duration across the frequency spectrum, and are more likely to cluster at lower frequencies. Similarly, Qian et al. [40] and Bruder et al. [41] found that pathological HFOs exhibit significantly higher amplitudes than physiological HFOs. Notably, the HFO characteristics identified in our study during active AE closely resemble those described in these prior investigations. An additional point of interest is the frequency shift observed in scalp HFOs during different EEG epochs in active AE. Specifically, HFOs during the interictal epoch exhibited lower average frequencies, whereas those during the ictal epoch showed markedly higher frequencies. The emergence of high-frequency HFOs (250–500 Hz), often referred to as fast ripples, has been shown to correlate temporally with seizure onset and is believed to reflect the formation of pathologically interconnected neuronal networks contributing to epileptogenesis [42,43]. Using an epilepsy mouse model, Khosravani et al. [44] quantified the temporal evolution of HFOs during the transition from interictal to ictal states. Their findings revealed a linear increase in low-frequency activity (0–100 Hz) approaching seizure onset, followed by a sharp rise in ripples and fast ripples (100–300 Hz) during the ictal transition. Similarly, studies using epileptic rats in a low Mg^2+^ model reported that higher-frequency HFO components (400–800 Hz) were predominantly observed in the preictal phase and at seizure onset. Taken together, these findings underscore the dynamic nature of HFOs and their potential role in seizure generation. Further research is warranted to characterize scalp HFOs more precisely and determine whether specific HFO profiles can serve as reliable markers of epileptogenic tissue.

This study has several limitations. First, to minimize artifacts, the scalp HFOs during the interictal epochs were evaluated mainly using sleep EEG recordings. As a result, the findings may not be directly generalizable to awake EEG recordings, which are more susceptible to artifacts such as muscle activity and movement, potentially leading to the detection of false HFOs [45]. Second, the relatively small sample size (*n* = 14) reflects the rarity of high-quality scalp EEG recordings with sufficient sampling rates for HFO analysis in pediatric absence epilepsy. While this limits generalizability, the study was designed as an exploratory investigation with strict artifact control and validated detection methods. Future multicenter studies with larger cohorts are warranted to confirm and expand upon these findings. Third, the EEG data in this study were recorded at a sampling frequency of 1024 Hz. Although this rate is sufficient to detect most HFOs within the ripple band (80–250 Hz), it does not fully meet the Nyquist criterion for capturing the entire upper-frequency range with optimal resolution; therefore, some HFOs may have been missed. Future studies employing higher sampling frequencies are warranted to confirm and refine the present findings.

## 4. Materials and Methods

### 4.1. Study Participants

We retrospectively identified children and adolescents (<18 years of age) who underwent scalp EEG at Fujita Health University Hospital between August 2022 and January 2025, using our institutional EEG database. Patients were included if they met the following criteria: (1) a diagnosis of childhood or juvenile AE according to the 2017 International League Against Epilepsy classification [46,47,48], and (2) scalp EEG recordings acquired with a high sampling frequency (≥1000 Hz). Among the enrolled participants, one child was excluded because of poor EEG recording quality, characterized by continuous artifacts affecting all electrodes. Another child was excluded because no scalp HFOs were detected. Ultimately, 12 children with childhood AE (4 males) and two adolescents with juvenile AE (1 male) were included in the study. The patients were categorized into the following two groups based on seizure control status: active AE and seizure-free AE. The active AE group included patients who exhibited absence seizures during the hyperventilation segment of the EEG and had ongoing clinical seizures, while the seizure-free AE group included patients who did not exhibit absence seizures during hyperventilation and were clinically seizure-free. Clinical data for each participant—including sex, ages at the time of the EEG recording and AE onset, seizure frequency, and medication history—were collected through a chart review.

This study was conducted in accordance with the principles of the Declaration of Helsinki and its subsequent amendments. It was approved by the Ethics Committee of Fujita Health University (Approval No. HM22–143). Patient consent was obtained using an opt-out procedure via the university website.

### 4.2. Scalp EEG Recordings

Scalp EEG recordings were obtained using the Neurofax system (Nihon Kohden, Tokyo, Japan), with 23 Ag/AgCl electrodes placed according to the international 10–20 system. A low-cut filter of 0.53 Hz was applied during data acquisition. EEG signals were sampled at a rate of 1024 Hz, and electrode impedances were maintained at ≤10 kΩ. Data analysis was conducted using the following channels in an average montage: Fp1, Fp2, F3, F4, C3, C4, P3, P4, O1, O2, F7, F8, T3, T4, T5, T6, Fz, Cz, and Pz. The electrode placement based on the international 10–20 system is illustrated in Supplemental Figure S1.

EEG recordings were performed following an initial calibration step and included commonly used standard activation procedures: photic stimulation, hyperventilation, and sleep. Hyperventilation was conducted for 3 or 5 min to provoke absence seizures.

### 4.3. Selection of EEG Recordings

EEG data selection was performed using the Neuro Workbench system (Nihon Kohden). EEG recordings were divided into three segments: (1) wakefulness, (2) sleep (Stages N1 and N2), and (3) hyperventilation. Both ictal and interictal epochs were annotated to assess the presence of scalp HFOs. Ictal epochs were identified within the hyperventilation segments. An ictal epoch was defined as the presence of regular 3-Hz generalized spike–wave discharges within the first second of seizure onset, consistent with absence seizures. The frequency range was set at 2.5–4 Hz for childhood absence epilepsy (CAE) and 3–5.5 Hz for juvenile absence epilepsy (JAE) [2]. A minimum seizure duration of 3 s was required for inclusion [5,49]. Interictal epochs were selected to be as artifact-free as possible, preferably during sleep and containing interictal epileptiform discharges (IEDs). IEDs were identified using a bipolar montage of unfiltered EEG displayed on a standard EEG interface. In this study, IEDs are referred to as “spikes”. Approximately 10 min of interictal EEG data per patient were reviewed.

EEG data selection was conducted by a specialist technician certified by the Japanese Society of Clinical Neurophysiology. The technician was blinded to the clinical characteristics of the patients, and the data were not used for clinical decision-making. EEG recordings were exported in European Data Format for subsequent scalp HFO analysis.

### 4.4. Detection of Scalp HFOs

Scalp HFOs were defined as oscillatory events comprising at least four cycles, with a center frequency within 80–250 Hz, clearly distinguishable from the background EEG signal [50]. EEG data from 19 channels were constructed using an average montage, and scalp HFO detection was performed across all channels. Initial detection of scalp HFOs was conducted automatically using an HFO detector, followed by visual verification by experienced reviewers. Automated detection was performed using the Hilbert Detector proposed by Crépon et al. [51], which has been clinically validated. The Hilbert Detector operates in two stages. In the first stage, EEG signals are band-pass filtered to isolate the frequency band of interest, and the signal envelope is computed using the Hilbert transform. In the second stage, local maxima—corresponding to potential scalp HFO events—are automatically identified using a threshold set at twice the SD of the envelope calculated over the entire EEG recording. All auto-detected HFOs were subsequently reviewed by a specialist technician certified by the Japanese Society of Clinical Neurophysiology. For the visual review of automatically detected HFOs, two independent reviewers evaluated the events. Inter-rater reliability was assessed using Cohen’s kappa coefficient and was confirmed to be above 0.8, indicating substantial agreement. Oscillatory events suspected to be noise or muscle artifacts were excluded. Detected scalp HFOs were classified into two categories: events superimposed on spikes, labeled as “spike ripples”, and events occurring independently of spikes, labeled as “ripples”. Previous studies have demonstrated that spike ripples are more specific biomarkers of the seizure onset zone than ripples alone [52]. Additionally, spike ripples are thought to reflect the burst firing of synchronized pyramidal neurons [33,35]. Therefore, in this study, we focused exclusively on spike ripples and excluded ripples from further analysis. Detection was performed blinded to the clinical data, and scalp HFOs were not used for clinical decision-making. All analyses were conducted using HFOApp [53] and MATLAB R2024a (MathWorks, Natick, MA, USA).

To characterize scalp HFOs, we calculated the average frequency, duration, amplitude, and number of cycles for each detected event. Frequency was defined as the rate of crest-to-trough oscillations within the HFO. Duration was defined as the time during which the Hilbert envelope remained above the detection threshold. Amplitude was defined as the highest peak-to-peak value, expressed as a z-score normalized to baseline. The number of cycles was calculated by dividing the event duration by the mean peak-to-peak interval.

### 4.5. K-Means Clustering Analysis

To investigate the distinct characteristics of scalp HFOs, k-means clustering analysis was performed. The key variables included average frequency, duration, and the log-transformed amplitude and number of cycles. Prior to clustering, we applied several preprocessing steps to improve the accuracy and stability of the algorithm. First, HFOs that exhibited extreme values in one or more features—clearly identified as outliers—were excluded from the analysis (*n* = 4). This step was essential because outliers can distort the position of cluster centroids and negatively affect clustering results. Second, amplitude values were log-transformed due to their skewed distribution and then standardized using z-score normalization. This was particularly important because amplitude is influenced by background EEG activity and tends to dominate distance calculations in clustering algorithms. Standardization ensured that all features contributed equally to the clustering process. The optimal number of clusters was determined using the elbow method. For each candidate cluster number, the total within-cluster sum of squares (WSS) was calculated, and a WSS curve was plotted. The “elbow” point—where the rate of decrease in WSS sharply changes—was considered indicative of the most appropriate number of clusters. In this study, the optimal number of clusters was determined to be three. This result is illustrated in Figure S2. Additionally, because k-means is a nondeterministic algorithm that can produce different results across runs as a result of random initialization, the clustering procedure was repeated approximately 300 times to ensure stability and reproducibility.

### 4.6. Statistical Analysis

First, k-means clustering was used to identify scalp HFO phenotypes with similar characteristics. Differences in scalp HFO characteristics between clusters were assessed using Tukey–Kramer’s honestly significant difference (HSD) test. Second, we hypothesized that clusters classified based on HFO characteristics would exhibit different distributions across three EEG epochs: (1) the ictal epoch in active AE, (2) the interictal epoch in active AE, and (3) the interictal epoch in seizure-free AE. To test this hypothesis, a two-tailed chi-square test was performed to compare the distribution of classified clusters across the three epochs. Additionally, multinomial logistic regression analysis was conducted to evaluate the relationship between cluster classification and EEG epoch type. Third, scalp HFO characteristics were compared across the three EEG epochs using Tukey–Kramer’s HSD test. Finally, multiple linear regression analysis was carried out to assess associations between scalp HFO characteristics and EEG epoch type. To clarify the statistical approach, we used multinomial logistic regression to evaluate the predictive value of HFO cluster classifications for EEG epoch types—specifically, to determine whether certain HFO phenotypes were more likely to occur during ictal or interictal states in active or seizure-free AE. Additionally, we applied multiple linear regression to assess the associations between individual HFO characteristics (average frequency, duration, amplitude, and number of cycles) and seizure states, allowing us to quantify how these features varied across different EEG epochs. Statistical significance was defined as a *p*-value < 0.05. All statistical analyses were performed using JMP software version 18 (SAS Institute Inc., Cary, NC, USA).

## 5. Conclusions

In this study, k-means clustering analysis was applied to scalp HFOs detected from EEG recordings in patients with AE based on four defining characteristics: average frequency, duration, amplitude, and number of cycles. This approach identified three distinct HFO clusters: Cluster 1, characterized by short duration and low amplitude; Cluster 2, characterized by low average frequency; and Cluster 3, characterized by long duration and high cycle count. To evaluate the clinical relevance of these HFO phenotypes, we examined their distribution across three EEG epochs: the ictal and interictal epochs in active AE, and the interictal epoch in seizure-free AE. Notably, Clusters 2 and 3 were significant predictors of ictal activity, while Cluster 2 was also significantly associated with interictal activity in active AE. A further comparison of HFO characteristics across the three EEG epochs revealed distinct physiological patterns: active AE was associated with scalp HFOs exhibiting lower average frequency and higher amplitude during the interictal epochs, and with HFOs showing higher frequency, longer duration, and greater cycle count during the ictal epochs. The three distinct scalp HFO phenotypes identified in this study may serve as novel biomarkers for seizure activity in AE. These findings suggest that scalp HFOs not only reflect underlying epileptiform activity, but also vary systematically by seizure state, thereby offering valuable insights into the dynamic neurophysiological mechanisms of AE. Although technical challenges remain in translating these findings into routine clinical practice, our findings offer a promising foundation for the development of EEG-based diagnostic tools tailored to the electrophysiological signatures of AE.

## Figures and Tables

**Table 1:** Patient characteristics stratified by seizure control status.

	Seizure-Free AE (*n* = 6)	Active AE (*n* = 8)	Total (*n* = 14)
Sex (*n*, %)			
Male	3 (21.4)	2 (14.3)	5 (35.7)
Female	3 (21.4)	6 (42.9)	9 (64.3)
Age at the time of EEG recording (years) ^1^	14.2 ± 3.0	9.0 ± 4.2	11.2 ± 4.5
Age at AE onset (years) ^1^	8.2 ± 2.9	6.5 ± 1.3	7.2 ± 2.2
Period of AE (day) ^1^	71.7 ± 43.1	29.1 ± 40.0	47.4 ± 45.3
Absence epilepsy type (*n*, %)			
CAE	4 (28.6)	8 (57.1)	12 (85.7)
JAE	2 (14.3)	0 (0.0)	2 (14.3)
Frequency of seizure (*n*, %)			
Rare	6 (42.8)	0 (0.0)	6 (42.8)
Yearly	0 (0.0)	2 (14.3)	2 (14.3)
Monthly	0 (0.0)	2 (14.3)	2 (14.3)
Daily/weekly	0 (0.0)	4 (28.6)	4 (28.6)
ASM (*n*, %)			
None	2 (14.5)	3 (21.4)	5 (35.9)
VPA	3 (21.4)	3 (21.4)	6 (42.8)
VPA + ESM	0 (0.0)	1 (7.1)	1 (7.1)
VPA + LEV	0 (0.0)	1 (7.1)	1 (7.1)
LCM + LTG	1 (7.1)	0 (0.0)	1 (7.1)
Analyzed EEG recording duration (seconds) ^2^			
Interictal epoch	600 (594.3–601.2)	600 (403.2–600.7)	600 (556.2–601.0)
Ictal epoch	–	12.0 (3.0–29.0)	12.0 (3.0–29.0)
Number of scalp HFOs detected (*n*, %)			
Interictal epoch	35 (21.5)	71 (43.5)	106 (65.0)
Ictal epoch	–	57 (35.0)	57 (35.0)

Abbreviations: AE, absence epilepsy; ASM, antiseizure medication; CAE, childhood absence epilepsy; EEG, electroencephalography; ESM, ethosuximide; HFO, high-frequency oscillation; JAE, juvenile absence epilepsy; LCM, lacosamide; LEV, levetiracetam; LTG, lamotrigine; VPA, valproate. ^1^ Data are presented as mean ± standard deviation (SD). ^2^ Data are presented as geometric mean with interquartile range (25th–75th percentiles).

**Table 2:** Multinomial logistic regression analysis of scalp high-frequency oscillation cluster groups as predictors of electroencephalogram epochs: the ictal and interictal epochs in active absence epilepsy (AE), and the interictal epoch in seizure-free AE.

					Multinomial Logistic Regression Analysis					
	Seizure-Free AE	Active AE			Interictal Epoch in Active AE vs. Interictal Epoch in Seizure-Free AE			Ictal Epoch in Active AE vs. Interictal Epoch in Seizure-Free AE		
	Interictal Epoch ^1^	Interictal Epoch ^1^	Ictal Epoch ^1^	*p* ^2^	OR	95% CI	*p*	OR	95% CI	*p*
Cluster 1	23 (35.4)	13 (20.0)	29 (44.6)	**<0.001**	Reference			Reference		
Cluster 2	10 (14.3)	53 (75.7)	7 (10.0)		2.71	1.23–5.67	**0.009**	0.33	0.14–0.74	**0.009**
Cluster 3	2 (7.1)	5 (17.9)	21 (75.0)		1.28	0.44–4.82	0.67	5.00	2.02–17.73	**0.002**

Abbreviations: AE, absence epilepsy; CI, confidence interval; OR, odds ratio. ^1^ Data are expressed as the number of HFOs and percentage (%). ^2^ *χ*^2^ test. *p* values < 0.05 are bolded and considered statistically significant.

**Table 3:** Multiple linear regression analysis of scalp high-frequency oscillation (HFO) characteristics across three electroencephalogram (EEG) epochs: the ictal and interictal epochs in active absence epilepsy (AE), and the interictal epoch in seizure-free AE.

Scalp HFO Characteristics	Crude				Adjusted for Age at EEG Recording			
	*β*	95% CI	Standardized *β*	*p*	*β*	95% CI	Standardized *β*	*p*
**Average frequency**								
Interictal epoch in seizure-free AE		Reference				Reference		
Interictal epoch in active AE	−16.19	−19.78–−12.61	−0.58	**<0.001**	−16.05	−21.56–−10.55	−0.58	**<0.001**
Ictal epoch in active AE	14.43	10.68–18.19	0.49	**<0.001**	14.58	8.76–20.41	0.50	**<0.001**
Age at EEG recording					0.06	−1.59–1.70	0.01	0.95
**Duration**								
Interictal epoch in seizure-free AE		Reference				Reference		
Interictal epoch in active AE	0.96	−2.14–4.06	0.05	0.54	0.52	−4.24–5.28	0.03	0.83
Ictal epoch in active AE	6.26	3.01–9.52	0.30	**<0.001**	5.79	0.75–10.84	0.28	**0.025**
Age at EEG recording					−0.17	−1.60–1.25	−0.04	0.81
**Log amplitude**								
Interictal epoch in seizure-free AE		Reference				Reference		
Interictal epoch in active AE	0.14	0.07–0.21	0.31	**<0.001**	0.19	0.08–0.30	0.42	**<0.001**
Ictal epoch in active AE	0.001	−0.07–0.08	0.003	0.97	0.06	−0.06–0.17	0.12	0.35
Age at EEG recording					0.02	−0.01–0.05	0.20	0.23
**Log number of cycles**								
Interictal epoch in seizure-free AE		Reference				Reference		
Interictal epoch in active AE	−0.08	−0.14–−0.02	0.53	**0.008**	−0.08	−0.17–0.007	−0.20	0.07
Ictal epoch in active AE	0.23	0.16–0.29	0.53	**<0.001**	0.22	0.13–0.32	0.52	**<0.001**
Age at EEG recording					−0.001	−0.03–0.03	−0.02	0.91

Abbreviations: AE, absence epilepsy; CI, confidence interval; EEG, electroencephalography; HFO, high-frequency oscillation. *p* values < 0.05 are bolded and considered statistically significant.

## Data Availability

The raw data supporting the conclusions of this article will be made available by the authors on request.

## Supplementary Materials

The following supporting information can be downloaded at: https://www.mdpi.com/article/10.3390/bioengineering13010065/s1, Figure S1: Electrode placement based on the international 10–20 system used for scalp EEG recordings in this study. This schematic illustrates the spatial configuration of electrodes including frontal (F), central (C), parietal (P), occipital (O), and temporal (T) regions, referenced to anatomical landmarks such as the nasion and inion. Figure S2: Determination of the optimal number of clusters using the elbow method. The plot shows the within-cluster sum of squares for different values of k in k-means clustering. The red dotted line indicates the inflection point (“elbow”), representing the optimal number of clusters selected for subsequent analysis.
