# Supplementary figures and images for: Phenotypic Classification of Scalp High-Frequency Oscillations in Absence Epilepsy Based on Multiple Characteristics Using K-Means Clustering
Source: Bioengineering (Basel). 2026 Jan 7;13(1):65. doi: 10.3390/bioengineering13010065 (PMC12837731; doi:10.3390/bioengineering13010065)

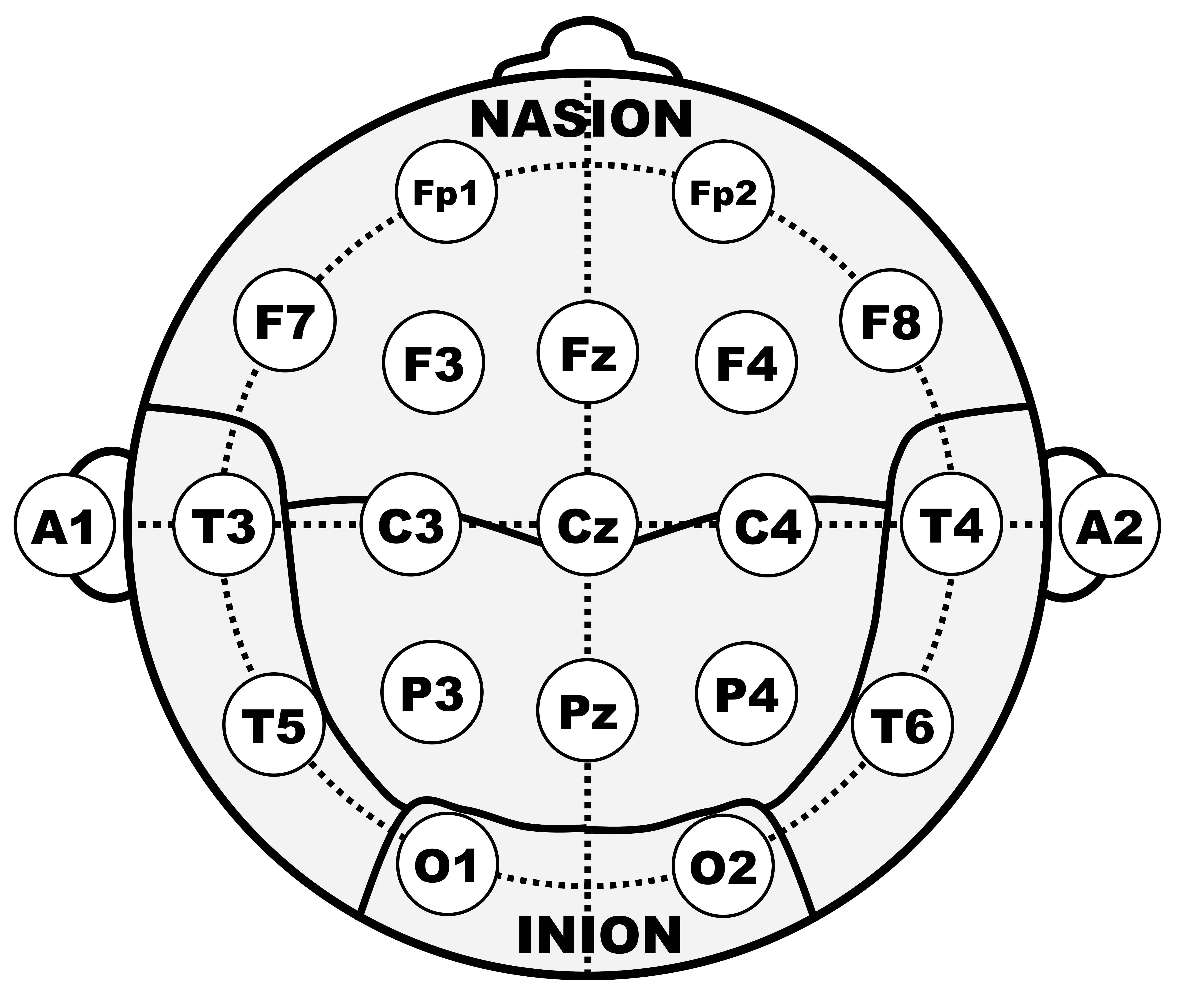

Supplement: Supplementary file 1 [file bioengineering-13-00065-s001.zip › Figure S1.png]

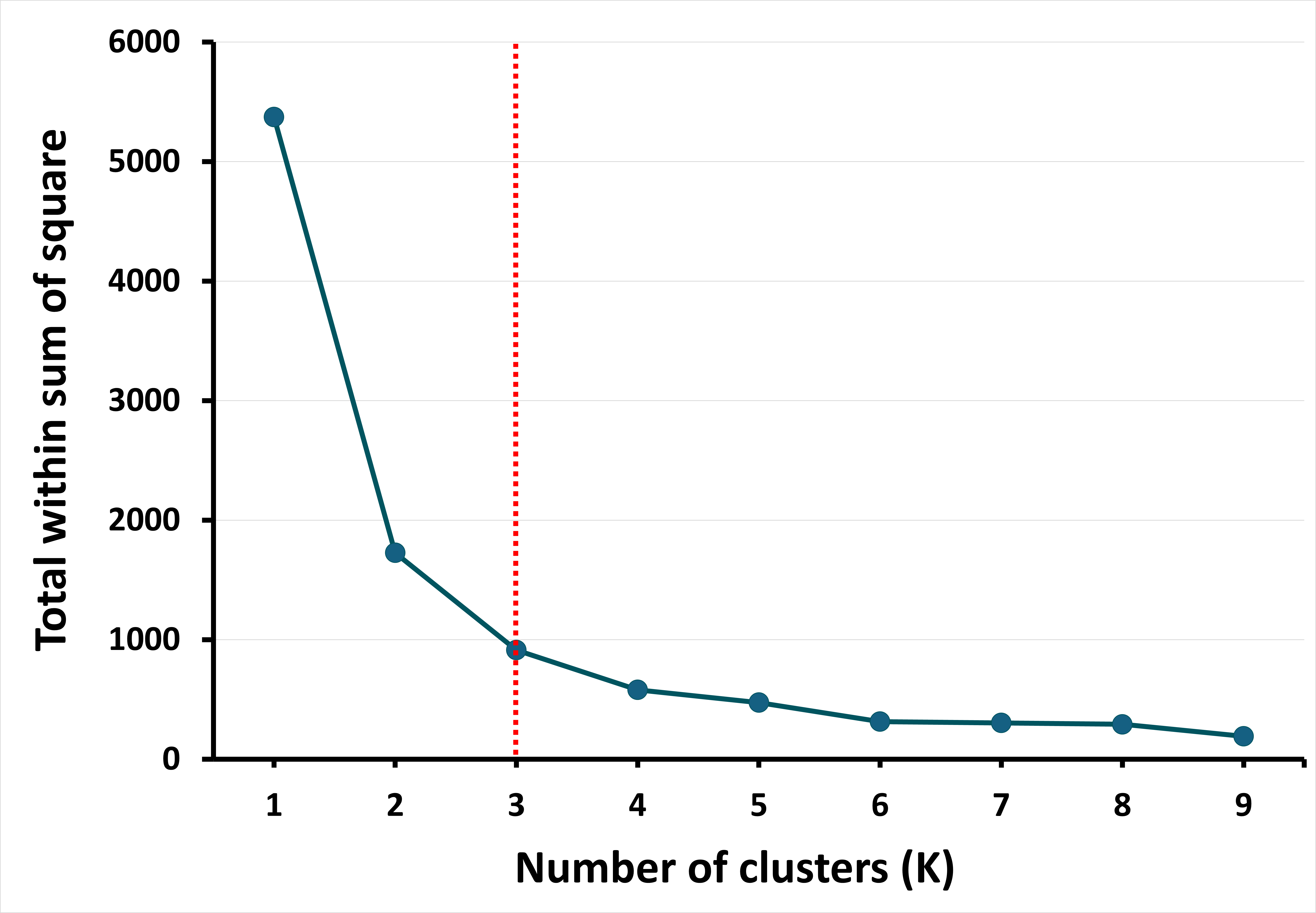

Supplement: Supplementary file 1 [file bioengineering-13-00065-s001.zip › Figure S2.png]
